# Herbaceous Peony (*Paeonia lactiflora* Pall.) as an Alternative Source of Oleanolic and Ursolic Acids

**DOI:** 10.3390/ijms12010655

**Published:** 2011-01-18

**Authors:** Chunhua Zhou, Ying Zhang, Yanle Sheng, Daqiu Zhao, Sansan Lv, Yue Hu, Jun Tao

**Affiliations:** College of Horticulture and Plant Protection, Yangzhou University, Yangzhou 225009, China; E-Mails: chzhou@yzu.edu.cn (C.Z.); 541981852@qq.com (Y.Z.); ylsheng2007@163.com (Y.S.); 405323488@qq.com (D.Z.); 309977018@qq.com (S.L.); 853089455@qq.com (Y.H.)

**Keywords:** Paeonia lactiflora, cultivar, oleanolic acid, ursolic acid, HPLC

## Abstract

Oleanolic acid (OA) and ursolic acid (UA) have been proven to possess many biological activities, and much attention is focused on the search for plants which are rich in OA and UA. In this report, the OA and UA accumulation characteristics were investigated in 47 cultivars of Chinese herbaceous peony (*Paeonia lactiflora* Pall.) and were followed in three cultivars over different developmental stages as measured by high performance liquid chromatography (HPLC). OA and UA levels in leaves and stems demonstrated an overall upward trend from May 1 to September 15 except for UA in the leaves of “Hong Feng”. The maximum values of OA and UA in leaves of “Yangfei Chu Yu”, “Fen Zhu Pan” and “Hong Feng” were 852.98, 575.60, 290.48 μg/g FW and 924.94, 827.36, 432.67 μg/g FW, respectively. The maximum values of OA and UA in stems of “Yangfei Chu Yu”, “Fen Zhu Pan” and “Hong Feng” were 359.28, 90.49, 43.90 μg/g FW and 326.86, 82.25, 56.63 μg/g FW, respectively. OA and UA contents in leaves of 47 different herbaceous peony cultivars ranged from 66.73–618.12 and 36.23–665.14 μg/g FW, respectively, with average values of 171.62 and 227.57 μg/g FW, respectively. The results suggested that the aboveground parts of herbaceous peony may be used as an alternative source of OA and UA for medicinal purposes in addition to its ornamental purposes.

## 1. Introduction

Herbaceous peony (*Paeonia lactiflora* Pall.), belonging to the Paeoniaceae family [1], is a perennial herb, and widely distributed in China. Herbaceous peony root is an important Traditional Chinese Medicine (TCM), and the flower of this plant is mainly used for ornamental purpose. Herbaceous peony is rich in different kinds of bioactive components, such as monoterpenes [2–4], volatile oil [5], triterpenes [6,7], tannins [8], stilbenes [9], flavonoids [7,10] and polyphenols [11,12], among which paeoniflorin—a kind of monoterpene—is the most studied medicinal compound [3,13–15]. During the cultivation of medicinal and ornamental herbaceous peony, aboveground biomass—mainly referring to the leaves and stems—is often discarded as litter, resulting in enormous waste of resources.

In recent years, more attention was paid to the isomeric pentacyclic triterpene oleanolic (OA) and ursolic acid (UA), which are usually widespread within the plant kingdom in the form of free state or glycosides. Many reports have shown that OA and UA mainly exist in *Chaenomeles sinensis* [16], *Crataegus pinnatifida* [17], *Diospyros kaki* [18,19], *Eriobotrya japonica* [20,21], *Eucalyptus globules* [22], *Ligustrum lucidum* [23], *Macrocarpium officinalis* [24], *Zizyphus jujuba* [25], and other species. Medical research has revealed that both OA and UA have many biological effects, such as antibacterial [26], anti-diabetic [27], anti-inflammatory [28], anti-tumor [29] and hepatoprotective activities [30]. Previous studies of herbaceous peony were mainly focused on the root [3,4,11,12,14,31], with monoterpenes as the main compounds. There were few reports about the characterization and quantification of OA and UA in the aboveground parts of herbaceous peony.

In this study, the OA and UA accumulation characteristics were investigated in 47 cultivars of Chinese herbaceous peony and specifically followed in three cultivars over different developmental stages based on high performance liquid chromatography (HPLC). The results may provide a theoretical basis for herbaceous peony as an alternative source of OA and UA.

## 2. Results

### 2.1. OA and UA Contents in Leaves and Stems of Herbaceous Peony at Different Developmental Stages

Herbaceous peony is usually divided into medicinal and ornamental types. Medicinal herbaceous peony is cultivated mainly for harvesting roots, while ornamental herbaceous peony is planted as cut flowers or pot plants. Therefore, investigation of OA and UA contents in herbaceous peony leaves and stems at different developmental stages, and determination of a reasonable harvest time, will be of great significance in ensuring comprehensive utilization of these resources.

#### 2.1.1. OA and UA Contents in Leaves of Herbaceous Peony at Different Developmental Stages

**Figure 1:**
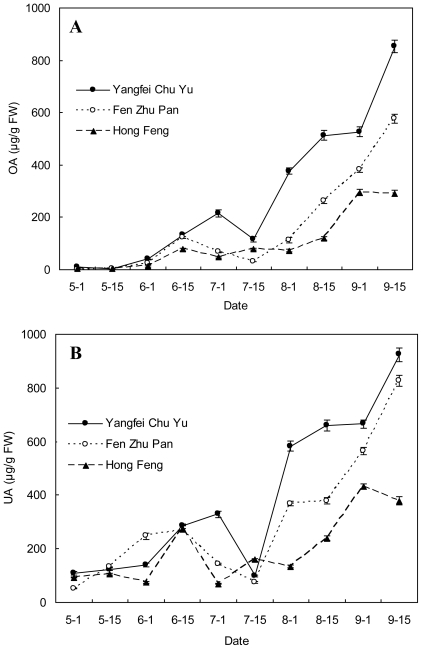
OA and UA contents (μg/g FW) in leaves of three herbaceous peony cultivars at different developmental stages. (**A**) OA content; (**B**) UA content.

The tendency for OA and UA in the leaves to change at different developmental stages is shown in Figure 1. The OA and UA contents changed little between May 1 and July 1 for all three cultivars, and a small difference were found between the cultivars. Then OA and UA contents sharply increased from July 15. In the following four developmental stages, significant differences between cultivars were observed, and the highest and lowest OA and UA contents were found in “Yangfei Chu Yu” and “Hong Feng”, respectively, with the OA and UA contents of “Fen Zhu Pan” being between those of the two other cultivars.

The changes of OA and UA contents in the leaves of herbaceous peony were generally similar among the three cultivars. The overall trend was upward from May 1 to September 15 with a small trough on July 15 for “Yangfei Chu Yu” and “Fen Zhu Pan”. For “Hong Feng”, the small trough of OA and UA in the leaves appeared earlier than the two other cultivars on July 1, with very little change in OA content, and by September 15, OA content maintained a relatively stable level, while the UA level even decreased.

The OA content in leaves of “Yangfei Chu Yu”, “Fen Zhu Pan” and “Hong Feng” was lowest on May 1, with 9.66, 2.76 and 0.94 μg/g FW, respectively. It reached the highest peaks on September 15, with 852.98, 575.60 and 290.48 μg/g FW, respectively, which was 88.30, 208.55 and 309.02 fold of the lowest value, respectively. The UA content in the leaves of “Yangfei Chu Yu”, “Fen Zhu Pan” and “Hong Feng” was also lowest on May 1, with 109.91, 93.30 and 51.96 μg/g FW, respectively. The highest UA content in leaves of “Yangfei Chu Yu” and “Fen Zhu Pan” appeared on September 15, with 924.94 and 827.36 μg/g FW, respectively, while those of “Hong Feng” reached the highest peak on September 1 with 432.76 μg/g FW. The highest UA content in leaves of “Yangfei Chu Yu”, “Fen Zhu Pan” and “Hong Feng” was 8.42, 8.87 and 8.33 fold of the lowest level, respectively.

**Figure 2:**
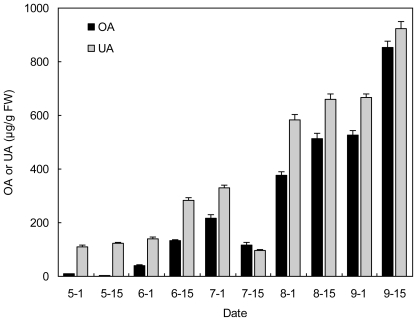
Comparison of OA and UA contents (μg/g FW) in leaves of herbaceous peony “Yangfei Chu Yu” at different developmental stages.

The ratio of UA to OA in the leaves obviously changed at the different developmental stages. For instance, the UA content in leaves of “Yangfei Chu Yu” was significantly higher (3.5–11.3 fold) than OA in the first three sampling times (May 1 to June 1) and then the ratio of UA to OA decreased to 1.1–2.1 fold, except for the samples on July 15 (Figure 2).

#### 2.1.2. OA and UA contents in Stems of Herbaceous Peony at Different Developmental Stages

**Figure 3:**
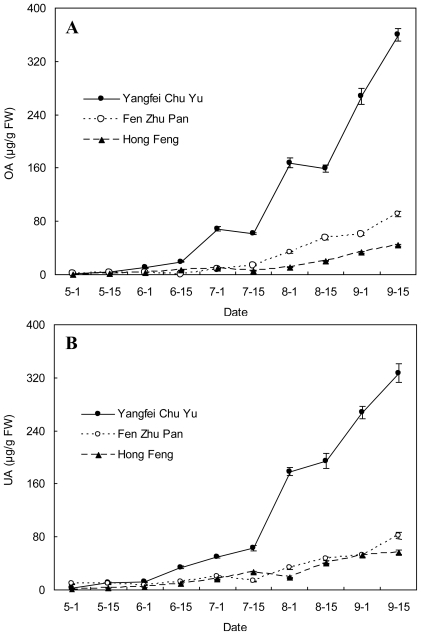
OA and UA contents (μg/g FW) in stems of three herbaceous peony cultivars at different developmental stages. (**A**) OA content; (**B**) UA content.

The OA and UA contents in stems of the three cultivars significantly changed at different developmental stages as shown in Figure 3. There was an overall upward trend of OA and UA contents from May 1 to September 15. The contents of OA and UA were lower than 20 μg/g FW before June 1 for all three cultivars. Then OA and UA contents in the stems of “Yangfei Chu Yu” accumulated more quickly than those of “Fen Zhu Pan” and “Hong Feng” from June 1, especially after July 15 with a more pronounced increase. For “Fen Zhu Pan” and “Hong Feng”, the OA content difference was very little before July 15, after which the content of OA in “Fen Zhu Pan” was higher than that in “Hong Feng”. However, the UA content of both cultivars had little difference during the whole development stage.

The OA content in stems of “Yangfei Chu Yu”, “Fen Zhu Pan” and “Hong Feng” was lowest on May 1, with 1.85, 1.89 and 0.25 μg/g FW, respectively. It reached the highest peaks on September 15, with 359.28, 90.49, and 43.90 μg/g FW, respectively, which was 194.21, 47.88 and 175.60 fold of the lowest value, respectively. The UA content in stems of “Yangfei Chu Yu”, “Fen Zhu Pan” and “Hong Feng” was also lowest on May 1, with 2.14, 8.88 and 0.49 μg/g FW, respectively. The highest UA content in stems of “Yangfei Chu Yu”, “Fen Zhu Pan” and “Hong Feng” appeared on September 15, with 326.86, 82.25 and 56.63μg/g FW, respectively, which was 152.74, 9.26 and 115.57 fold of the lowest level, respectively.

**Figure 4:**
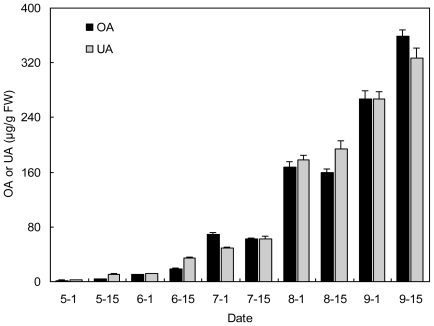
Comparison of OA and UA contents (μg/g FW) in stems of herbaceous peony “Yangfei Chu Yu” at different developmental stages.

Very little difference in both OA and UA content was observed in the stems as compared with leaves. For instance, the ratio of UA to OA in stems in “Yangfei Chu Yu” was 0.9–2.6 from May 1 to September 15 (Figure 4).

### 2.2. OA and UA Contents in Leaves of Different Herbaceous Peony Cultivars

Great differences existed between the OA and UA contents of leaves of 47 investigated herbaceous peony cultivars. The contents of OA and UA were 54.26–618.12 (average value 171.62) and 36.23–665.14 (average value 227.57) μg/g FW, respectively. The highest contents of OA and UA in leaves were 11.39 and 18.34 fold of the lowest levels, respectively. Among 47 cultivars, “Hong Xiu Qiu” and “Yangfei Chu Yu” contained the highest OA and UA contents, with 618.12 and 665.14 μg/g FW, respectively, while the lowest OA and UA contents were observed in “Fen Zi Lu Jin” and “Jiangshan Ru Hua”, with 54.26 and 36.23 μg/g FW, respectively (Table 1).

The UA content in leaves was generally higher than OA with some exceptions among the 47 cultivars. OA content was mainly concentrated between 100–200 μg/g FW, while the UA content was more than 200 μg/g FW in over half the cultivars. The ratio of UA to OA in leaves of the different herbaceous peony cultivars ranged from 0.52–3.72, with an average value of 1.43.

**Figure 5:**
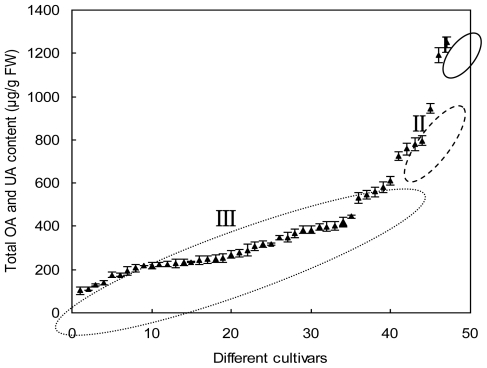
Distribution of the total OA and UA contents (μg/g FW) in leaves of different herbaceous peony cultivars. I, high-content; II, medium-content; III, low-content.

Similarly, the total OA and UA content in leaves of the different cultivars also largely differed with a range of 102.60–1251.38 μg/g FW (Table 1). From the distribution plot of total amount of OA and UA in leaves (Figure 5), herbaceous peony can be divided into high- (>1000 μg/g FW), medium- (700–1000 μg/g FW) and low-content (<700 μg/g FW) types. Two cultivars, “Hong Xiu Qiu” and “Yangfei Chu Yu”, belonged to high-content type, with values of 1251.38 and 1194.08 μg/g FW, respectively. The medium-content type included five cultivars, “Fen He Piao Xiang”, “Xishi Fen”, “Hong Ling”, “Fen Zhu Pan” and “Hong Feng”, with an average content of 801.18 μg/g FW. The remaining 40 cultivars were classified as low-level type, with an average concentration of 307.83 μg/g FW.

## 3. Discussion

### 3.1. OA and UA Distribution in Herbaceous Peony at Different Development Stages

Lu *et al.* [32] found that the UA content of old leaves (1.21%) of Guangxi Kudingcha was higher than that of young leaves (0.57%). Zou [33] reported that OA and UA levels in ripened fruits of *Forsythia suspensa* (0.5490% and 0.406%) were significantly higher than those in young (0.3805% and 0.3275%) and mature fruits (0.417% and 0.346%). Our study indicated that OA and UA contents of old leaves and stems of herbaceous peony were also higher than those of young leaves and stems. Therefore, a mode of OA and UA content change may exist in plants such that the contents of OA and UA in old organs are higher than those in young ones during development.

### 3.2. OA and UA Distribution in Different Cultivars of Herbaceous Peony

The contents of OA and UA in the same part of different cultivars differed in varied degrees. Wang *et al.* [34] determined OA and UA contents in fruits of jujube cultivars and wild jujube, and the results showed that the contents in “Fupingdazao”, “Junzao” and “Yazao” were higher than those in “Sanbianhong”, “Popozao”, “Jianzao” and “Jinsixiaozao”. The research results of Zhou *et al*. [21] demonstrated that OA and UA contents in flower of five loquat cultivars, “Dahongpa”, “Baozhu”, “Dayeyangdun”, “Jiajiao” and “Ruantiaobaisha”, differed slightly, with the highest UA and OA contents in “Dahongpa”, and the lowest UA and OA contents in “Ruantiaobaisha”. In our study, the data demonstrated that great differences existed in UA and OA contents of the leaves between different herbaceous peony cultivars. The contents of OA and UA were 54.26–618.12 (171.62) and 36.23–633.26 (227.57) μg/g FW, respectively. According to the total amount of OA and UA in leaves, herbaceous peony can be divided into high- (>1000 μg/g FW), medium- (700–1000 μg/g FW) and low-content (<700 μg/g FW) types, and about five-sixths of the cultivars belonged to the low-content type.

There were some reports about the relationship between flower color and the OA and UA contents in plants. Huang [35] found significant differences in OA and UA content of azalea flower between cultivars of different color. The pink flower had higher OA and UA contents, white flower had lower OA and UA contents, and OA and UA contents in red flower were between the two cases of pink and white flowers. In our study, OA and UA levels in leaves of herbaceous peony may have some correlation with flower color. In general, triterpenes was relatively low in the cultivars with deep color (purple) flowers, while they were relatively high in the cultivars with pale color (pink, pale pink, white) flowers, which may be related to the balance of secondary metabolism in plants. However, further study is needed to assess whether flower color is closely related to OA and UA contents.

### 3.3. Comparison of OA and UA Contents in Herbaceous Peony with Other Plants

OA and UA are widely distributed in plants with great variations between different families, genera and species. The results of our experiment showed that the contents of OA and UA in herbaceous peony leaves were in the range of 54.26–618.12 μg/g (0.0054%–0.0618% FW) and 36.32–633.26 μg/g (0.0036%–0.0633%) based on fresh weight, respectively. Considering the roughly 90% water content of plant leaves, OA and UA contents in dry leaves would increase ten-fold over that in fresh samples, with about 0.054%–0.618% and 0.036%–0.633%, respectively; these levels are higher than or similar to those of *Callicarpa nudiflora* leaves [36], *Pterocephalus hookerie* underground and aboveground parts [37], *Hedyotis diffusa* whole plant [38], *Fructus chaenomeles* fruits [39], and *Ligustrum lucidum* fruits [40].

## 4. Experimental Section

### 4.1. Chemicals

OA (TCM-031, purity ≥ 98%) and UA (TCM-036, purity ≥ 98%) standards were purchased from Nanjing TCM Institute of Chinese Materia Medica (Nanjing, Jiangsu, China). HPLC grade methanol was obtained from Caledon Laboratories Co. (Georgetown Ont., Canada). The deionized distilled water (ddH_2_O) was obtained with a Milli-Q water purification system from Millipore (Bedford, MA). All other reagents used in the present study were of analytical grade.

### 4.2. Plant Materials

Herbaceous peony (*Paeonia lactiflora* Pall.) was planted in the Germplasm Nursery of Herbaceous Peony at College of Horticulture and Plant Protection, Yangzhou University, Jiangsu Province, China (32°30′N, 119°25′E). The leaves and stems of “Yangfei Chu Yu”, “Fen Zhu Pan” and “Hong Feng” were picked every 15 days from May 1 to September 15, 2009. The leaves of 47 cultivars were collected on September 1, 2009. All the samples were frozen in liquid nitrogen immediately after taking back to laboratory, and then stored in refrigerator at −20 °C until analysis.

### 4.3. Extraction

For preparation of crude extract, a suitable amount of plant materials of each sample was collected and fully grinded with liquid nitrogen. Subsequently, 2 g of sample was weighed and transferred to a 10 mL centrifuge tube, and 5 mL ethanol was added in and mixed homogeneously, and then extracted for 30 min with KQ-500B ultrasonic cleaning machine (Kunshan Ultrasonic Equipment Co. Ltd., Kunshan, Jiangsu, China). The sample was centrifuged at 8000 g for 10 min, and then the filtrate was collected. Precipitates were extracted again and both filtrates were pooled and evaporated to dryness at 35 °C. The residue was dissolved in 1.5 mL methanol and transferred to a 2 mL tube. The crude extract was filtered through a 0.22 μm micro-filter before HPLC analysis. Each sample was repeated in triplicate.

### 4.4. HPLC Analysis

Quantifications of OA and UA were performed on a Waters-2695 HPLC system (Beckman coulter, USA) equipped with Waters 2695 pump and 2487 UV detector according to the method of Zhou *et al*. [20] with some modifications. The column was a Lichrospher C18 (4.6 × 250 mm, 5 μm) column (Hanbon Sci. and Tech, China) equipped with a guard column with the same stationary phase. All these two compounds were detected at 210 nm at room temperature with a flow rate of 1.0 mL/min and an injection volume of 20 μL. The mobile phase consisted of methanol (A) and 0.1% phosphoric acid solution (B) with a ratio of 85:15 (A:B, v/v) for simultaneous detection of OA and UA. The contents of both compounds were calculated according to the standard curves obtained with a series of standard solutions (0, 25, 50, 75, and 100 μg/mL).

### 4.5. Statistical Analysis

All data are means of three replicates with standard deviations. Microsoft Excel (Microcal Software Inc., Northampton, MA, USA) was used to calculate standard deviations.

## 5. Conclusions

Traditional cultivation of both medicinal and ornamental herbaceous peony often causes a serious waste of resources due to discarding the aboveground biomasses. For medicinal purpose, herbaceous peony roots are usually harvested in August or September. During the same period, the aboveground parts of ornamental herbaceous peony will complete assimilate transportation and start to wilt in August to September. The OA and UA contents in the aboveground parts, mainly including the stems and leaves, reached a higher level from August to September. Therefore, the aboveground parts of herbaceous peony may be used as an alternative medicinal source of OA and UA in addition to its ornamental purposes.

## Figures and Tables

**Table 1:** OA and UA contents (μg/g FW) in leaves of different herbaceous peony cultivars.

No.*	Cultivar	Flower Color	OA	UA	OA + UA
**1**	Jiangshan Ru Hua	Purple	66.37 ± 7.44	36.23 ± 3.78	102.60 ± 9.93
**2**	Fen Zi Lu Jin	Purple	54.26 ± 4.53	57.38 ± 5.07	111.64 ± 9.22
**3**	Shuang Hong Lou	Purple	69.57 ± 8.57	59.48 ± 2.32	129.05 ± 6.26
**4**	Zi Pao	Purple	68.89 ± 7.27	69.75 ± 4.36	138.64 ± 10.49
**5**	Hong Pan Xiang Cui	Purple	87.63 ± 12.15	88.14 ± 1.99	175.77 ± 12.82
**6**	Zi Pan Tuo Jin	Pink	97.42 ± 4.63	78.63 ± 5.64	176.05 ± 9.12
**7**	Jin Xing Can Lan	Pink	107.00 ± 12.91	87.48 ± 8.38	194.48 ± 21.29
**8**	Zi Yun Feng Bo	Pink	134.54 ± 8.90	71.96 ± 9.40	206.50 ± 17.58
**9**	Fen Lan Lou	Pink	96.11 ± 10.17	121.17± 10.57	217.28 ± 1.29
**10**	Chun Xiao	Pink	76.34 ± 3.72	141.16 ± 11.03	217.50 ± 14.75
**11**	Yu Lou Hong Xing	Pink	130.18 ± 11.22	93.13 ± 10.98	223.31 ± 8.72
**12**	Lian Die	Purple	87.63 ± 5.07	137.74 ± 6.00	225.37 ± 11.03
**13**	Hong Yan Zheng Hui	Purple	72.54 ± 7.71	157.43 ± 11.34	229.97 ± 19.04
**14**	Fen Lan Xiu Qiu	Pink	110.67 ± 11.12	122.22 ± 8.03	232.89 ± 16.09
**15**	Zi Yan	Purple	103.75 ± 10.04	130.41 ± 9.53	234.16 ± 2.63
**16**	Jin Zan Ci Hhong Ling	Pink	96.87 ± 7.98	145.94 ± 12.70	242.81 ± 20.64
**17**	Fen Ling Jiao Yan	Pink	139.03 ± 6.15	106.81 ± 9.70	245.84 ± 15.80
**18**	Zi Lou	Purple	113.55 ± 9.61	136.35 ± 8.82	249.90 ± 18.24
**19**	Jin Shang Ttian Hua	Purple	99.00 ± 7.74	154.76 ± 13.15	253.76 ± 19.59
**20**	Zi Hong Jian Rong	Pink	132.82 ± 6.92	135.99 ± 11.41	268.81 ± 18.00
**21**	Fen Yu Long	Pink	108.85 ± 4.60	166.83 ± 12.11	275.68 ± 16.46
**22**	Fen Xiang Lou	Deep pink	93.14 ± 11.70	195.34 ± 16.15	288.48 ± 27.81
**23**	Xin Hua Nu Fang	Pink	122.05 ± 7.80	185.39 ± 11.70	307.44 ± 19.46
**24**	Fu Gui Hong	Deep pink	112.28 ± 3.72	204.09 ± 11.59	316.37 ± 15.03
**25**	Wan Zhuang Fen	Pink	133.54 ± 8.97	185.09 ± 10.89	318.63 ± 4.34
**26**	Da Fu Gui	Pink	130.68 ± 4.94	215.74 ± 11.56	346.42 ± 8.58
**27**	Huang Jin Si	Pink	104.44 ± 7.45	243.75 ± 14.51	348.19 ± 21.94
**28**	Zhong Sheng Fen	Pale pink	180.38 ± 8.53	187.40 ± 12.66	367.78 ± 20.35
**29**	Fen Yu Qiu	Pink	167.38 ± 3.55	215.13 ± 13.83	382.51 ± 17.35
**30**	Hong Xiu Zhen	Pink	97.70 ± 8.84	286.80 ± 10.27	384.50 ± 19.08
**31**	Zi Rong Ji Yao	Pink	206.75 ± 8.48	190.21 ± 7.59	396.96 ± 15.74
**32**	Pan Tuo Shan Hu	Pink	192.57 ± 11.03	207.00 ± 13.21	399.57 ± 22.33
**33**	Jin Hui	Pink	178.91 ± 9.41	222.27 ± 10.37	401.18 ± 19.78
**34**	Hong Xian Xiu Yu	Yellow white	147.51 ± 8.15	271.27 ± 14.98	418.78 ± 22.31
**35**	Lian Hua Zan	Purple	197.29 ± 6.35	247.15 ± 12.16	444.44 ± 5.91
**36**	Hong Lou Piao Xian	Purple	214.23 ± 12.83	315.98 ± 12.58	530.21 ± 25.11
**37**	Lan Cui Qiu	Purple	221.23 ± 13.01	325.69 ± 8.15	546.92 ± 20.80
**38**	Man Tang Hong	Purple	153.57 ± 10.44	405.04 ± 13.97	558.61 ± 24.39
**39**	Hai Tang Ji Jin	Pink	274.94 ± 14.41	306.95 ± 10.57	581.89 ± 24.96
**40**	Di Lian Qi Hua	Pale pink	173.51 ± 6.90	438.25 ± 13.29	611.76 ± 20.09
**41**	Hong Feng	Purple	295.20 ± 11.77	431.76 ± 10.20	726.96 ± 20.13
**42**	Fen He Piao Xiang	Pale pink	333.07 ± 12.94	425.43 ± 13.80	758.50 ± 26.66
**43**	Xishi Fen	Pale pink	344.85 ± 14.40	434.89 ± 15.14	779.74 ± 29.36
**44**	Hong Ling	Pink	402.11 ± 10.21	393.68 ± 11.36	795.79 ± 21.55
**45**	Fen Zhu Pan	Pink	380.84 ± 8.92	564.10 ± 14.17	944.94 ± 22.95
**46**	Yangfei Chu Yu	White	525.95 ± 18.97	665.14 ± 15.70	1191.08 ± 34.63
**47**	Hong Xiu Qiu	Pink	618.12 ± 8.00	633.26 ± 15.54	1251.38 ± 23.50

* Cultivars are arranged in order starting from that with the lowest total OA and UA content, to the highest level.

## References

[1] Hong DY Pan KY Paeoniaceae Flora of China Wu ZY Raven PH Science Press and Missouri Botanic Garden Press Beijing, China 2001 6 127 132

[2] Braca A Kiem PV Yen PH Nhiem NX Quang TH Cuong NX Minh CV New monoterpene glycosides from *Paeonia lactiflora* Fitoterapia 2008 79 2 117 120 18201840 10.1016/j.fitote.2007.11.001

[3] Kim N Park KR Park IS Park YH Application of novel HPLC method to the analysis of regional and seasonal variation of the active compounds in *Paeonia lactiflor* Food Chem 2006 96 3 496 502

[4] Wang HB Gu WF Chu WJ Zhang S Tang XC Qin GW Monoterpene glucosides from *Paeonia lactiflor* J Nat Prod 2009 72 7 1321 1324 19402674 10.1021/np8001783

[5] Kumar N Motto MG Volatile constituents of peony flowers Phytochemistry 1985 25 1 250 253

[6] Ikuta A Kamiya K Satake T Saiki Y Triterpenoids from callus tissue cultures of *Paeonia* species Phytochemistry 1995 38 5 1203 1207

[7] Kamiya K Yoshioka K Saiki Y Ikuta A Satake T Triterpenoids and flavonoids from *Paeonia lactiflor* Phytochemistry 1997 44 1 141 144

[8] Tanaka T Fukumori M Ochi T Kouno I Paeonianins A−E, New dimeric and monomeric ellagitannins from the fruits of *Paeonia lactiflora* J Nat Prod 2003 66 6 759 763 12828458 10.1021/np020608g

[9] Kim HJ Chang EJ Bae SJ Shim SM Park HD Rhee CH Park JH Choi SW Cytotoxic and antimutagenic stilbenes from seeds of *Paeonia lactiflor* Arch Pharm Res 2002 25 3 293 299 12135100 10.1007/BF02976629

[10] Jia N Shu QY Wang LS Du H Xu YJ Liu ZA Analysis of petal anthocyanins to investigate coloration mechanism in herbaceous peony cultivars Sci Hortic 2008 117 2 167 173

[11] Guo D Ye G Guo H A new phenolic glycoside from *Paeonia lactiflor* Fitoterapia 2006 77 7 8 613 614 10.1016/j.fitote.2006.05.026 16905278

[12] Lee SC Kwon YS Son KH Kim HP Heo MY Antioxidative constituents from *Paeonia lactiflor* Arch Pharm Res 2005 28 7 775 783 16114491 10.1007/BF02977342

[13] Chen F Lu HT Jiang Y Purification of paeoniflorin from *Paeonia lactiflora* Pall. by high-speed counter-current chromatography J Chromatogr A 2004 1040 2 205 208 15230527 10.1016/j.chroma.2004.04.023

[14] Lee B Shin YW Bae EA Han SJ Kim JS Kang SS Kim DH Antiallergic effect of the root of *Paeonia lactiflora* and its constituents paeoniflorin and paeonol Arch Pharm Res 2008 31 4 445 450 18449501 10.1007/s12272-001-1177-6

[15] Xiao L Wang YZ Liu J Luo XT Ye Y Zhu XZ Effects of paeoniflorin on the cerebral infarction, behavioral and cognitive impairments at the chronic stage of transient middle cerebral artery occlusion in rats Life Sci 2005 784 12 413 420 16137717 10.1016/j.lfs.2005.04.069

[16] Fang XS Wang JH Yu XL Zhang GH Zhao JP Optimization of microwave-assisted extraction followed by RP-HPLC for the simultaneous determination of oleanolic acid and ursolic acid in the fruits of *Chaenomeles sinensi* J Sep Sci 2010 33 8 1147 1155 20183824 10.1002/jssc.200900726

[17] Cui T Li JZ Kayahara H Ma L Wu LX Nakamura K Quantification of the polyphenols and triterpene acids in Chinese hawthorn fruit by high-performance liquid chromatography J Agric Food Chem 2006 54 13 4574 4581 16787000 10.1021/jf060310m

[18] Fan JP Zhang RF Zhu JH Optimization of microwave assisted extraction of total triterpenoid in *Diospyros kaki* leaves using response surface methodology Asian J Chem 2010 22 5 3487 3500

[19] Zhou CH Sheng YL Zhao DQ Wang ZQ Tao J Variation of oleanolic and ursolic acid in the flesh of persimmon fruit among different cultivars Molecules 2010 15 9 6580 6587 20877245 10.3390/molecules15096580 PMC6257790

[20] Ho HY Lin WC Kitanaka S Chang CT Wu JB Analysis of bioactive triterpenes in *Eriobotrya japonica* Lindl. by high-performance liquid chromatography J Food Drug Anal 2008 16 6 41 45

[21] Zhou CH Chen KS Sun CD Chen QJ Zhang WS Li X Determination of oleanolic acid, ursolic acid, and amygdalin in the flower of *Eriobotrya japonica* Lindl. by HPLC Biomed Chromatogr 2007 21 7 755 761 17385800 10.1002/bmc.817

[22] Domingues RMA Sousa GDA Freire CSR Silvestre AJD Pascoal Neto C *Eucalyptus globulus* biomass residues from pulping industry as a source of high value triterpenic compounds Ind Crop Prod 2010 31 1 65 70

[23] Liu HX Shi YH Wang DX Yang GL Yu AM Zhang HQ MECC determination of oleanolic acid and ursolic acid isomers in *Ligustrum lucidum* Ait J Pharm Biomed Anal 2003 32 3 479 485 14565552 10.1016/s0731-7085(03)00235-8

[24] Wang H Wang Z Guo W Comparative determination of ursolic acid and oleanolic acid of *Macrocarpium officinalis* (Sieb. et Zucc.) Nakai by RP-HPLC Ind Crop Prod 2008 28 3 328 332

[25] Guo S Duan JA Tang YP Su SL Shang EX Ni SM Qian DW High-performance liquid chromatography-Two wavelength detection of triterpenoid acids from the fruits of *Ziziphus jujuba* containing various cultivars in different regions and classification using chemometric analysis J Pharm Biomed Anal 2009 49 5 1296 1302 19359121 10.1016/j.jpba.2009.03.006

[26] Wolska KI Grudniak AM Fiecek B Kraczkiewicz-Dowjat A Kurek A Antibacterial activity of oleanolic and ursolic acids and their derivatives Cent Eur J Biol 2010 5 5 543 553

[27] Gutierrez RMP Solis RV Baez EG Navarro YG Hypoglycemic activity of constituents from *Astianthus viminalis* in normal and streptozotocin-induced diabetic mice J Nat Med 2009 63 4 393 401 19484331 10.1007/s11418-009-0343-7

[28] Huang LJ Gao WY Li X Zhao WS Huang LQ Liu CX Evaluation of the *in vivo* anti-inflammatory effects of extracts from *Pyrus bretschneideri* Rehd J Agric Food Chem 2010 58 16 8983 8987 10.1021/jf101390q 20672838

[29] Yan SL Huang CY Wu ST Yin MC Oleanolic acid and ursolic acid induce apoptosis in four human liver cancer cell lines Toxicol in Vitro 2010 24 3 842 848 20005942 10.1016/j.tiv.2009.12.008

[30] Shyu MH Kao TC Yen GC Oleanolic acid and ursolic acid induce apoptosis in HuH7 human hepatocellular carcinoma cells through a mitochondrial-dependent pathway and downregulation of XIAP J Agric Food Chem 2010 58 10 6110 6118 20415421 10.1021/jf100574j

[31] Baumgartner RR Steinmann D Heiss EH Atanasov AG Ganzera M Stuppner H Dirsch VM Bioactivity-guided isolation of 1,2,3,4,6-Penta-*O*-galloyl-d-glucopyranose from *Paeonia lactiflora* roots as a PTP1B inhibitor J Nat Prod 2010 73 9 1578 1581 20806783 10.1021/np100258e

[32] Lu M Meng DP Rong YP Comparison of ursolic acid contents between the young and old leaves of Guangxi Kudingcha (in Chinese) Chin J Exp Tradit Med Form 2008 14 10 14 15

[33] Zou SQ RP-HPLC determination of ursolic acid and oleano1ic acid in various collecting period of *Forsythia suspensa* (Thunb.) Vahl. (in Chinese) J Shenenyang Pharm Univ 2007 24 3 164 166 171

[34] Wang XH Cui T Qi XJ Du GS Zhao J Liu MJ Determination of oleanolic and ursolic acid contents in jujube and wild jujube fruit by HPLC (in Chinese) Chin J Food Sci 2002 23 6 137 138

[35] Huang HW Content comparison of ursolic acid and oleanolic acid in different color flowers of *Rhododendron simsii* Planch (in Chinese) Life Sci Instr 2008 6 12 16 19

[36] Zhang YQ Hong JB Liu WL Determination of oleanolic acid and ursolic acid in the leaves of *Callicarpa nudiflorea* Hook. Et Arn. by RP-HPLC (in Chinese) J Hainan Med College 2009 15 1 5 7

[37] Feng YJ Zhang Y Determination of oleanolic acid and ursolic acid in *Pterocephalus hookerie* (C.B. Clarke) Hook by HPLC-ELSD (in Chinese) J Chengdu Univ TCM 2007 30 3 54 56

[38] Zhang CH Guo XJ Li F Xue XF Li MF HPLC determination of the contents of oleanolic acid and ursolic acid in whole plant of *Hedyotis diffusa* Willd. (in Chinese) J Shenyang PharmUniv 2004 21 5 358 360 370

[39] Wang DJ Wang X Geng YL Li SB Determination of oleanolic acid and ursolic acid in the fruit of different *Fructus chaenomeles* cultivars by RP-HPLC (in Chinese) Food Sci 2008 29 10 497 499

[40] Li FM Zhu CC Determination of oleanolic acid and ursolic acid in the fruits of *Ligustrum lucidum* Ait. by RP-HPLC (in Chinese) Pharm Today 2009 19 4 37 40

